# Engineering of Liposome Structure to Enhance Physicochemical Properties of *Spirulina plantensis* Protein Hydrolysate: Stability during Spray-Drying

**DOI:** 10.3390/antiox10121953

**Published:** 2021-12-06

**Authors:** Maryam Mohammadi, Hamed Hamishehkar, Marjan Ghorbani, Rahim Shahvalizadeh, Mirian Pateiro, José M. Lorenzo

**Affiliations:** 1 Drug Applied Research Center, Student Research Committee, Tabriz University of Medical Sciences, Tabriz 51656-65811, Iran; rahimshahvalizadeh@gmail.com; 2 Department of Food Science, Faculty of Agriculture, University of Tabriz, Tabriz 51666-16471, Iran; 3 Drug Applied Research Center, Tabriz University of Medical Sciences, Tabriz 51656-65811, Iran; hamishehkarh@tbzmed.ac.ir; 4 Stem Cell Research Center, Tabriz University of Medical Sciences, Tabriz 51666-14766, Iran; Ghorbani.marjan65@yahoo.com; 5 Centro Tecnológico de la Carne de Galicia, Avd. Galicia No. 4, Parque Tecnológico de Galicia, 32900 Ourense, Spain; jmlorenzo@ceteca.net; 6 Facultade de Ciencias, Universidade de Vigo, Área de Tecnoloxía dos Alimentos, 32004 Ourense, Spain

**Keywords:** *Spirulina platensis*, bioactive peptides, encapsulation, liposomes, chitosome

## Abstract

Encapsulating hydrolysates in liposomes can be an effective way to improve their stability and bioactivity. In this study, *Spirulina* hydrolysate was successfully encapsulated into nanoliposomes composed of different stabilizers (cholesterol or γ-oryzanol), and the synthesized liposomes were finally coated with chitosan biopolymer. The synthesized formulations were fully characterized and their antioxidant activity evaluated using different methods. Then, stabilization of coated nanoliposomes (chitosomes) by spray-drying within the maltodextrin matrix was investigated. A small mean diameter and homogeneous size distribution with high encapsulation efficiency were found in all the formulations, while liposomes stabilized with γ-oryzanol and coated with chitosan showed the highest physical stability over time and preserved approximately 90% of their initial antioxidant capacity. Spray-dried powder could preserve all characteristics of peptide-loaded chitosomes. Thus, spray-dried hydrolysate-containing chitosomes could be considered as a functional food ingredient for the human diet.

## 1. Introduction

The microalga *Spirulina* has gained more attention in areas such as the pharmaceutical, food, poultry, and aquaculture industries for its nutritional and health benefits [1]. Certain therapeutic effects of *Spirulina* (reduced hyperlipidemia, obesity, and blood cholesterol; antioxidant and anticancer activity; immune system improvement; and increased beneficial intestinal bacteria) have been proven by pre-clinical and clinical studies, which are related to their bioactive constitution, e.g., phycocyanins, carotenoids, phenolic compounds, and polyunsaturated fatty acids. The green-blue microalgae are a rich source of proteins (60–70% of dry matter) and, due to the absence of cellulose in the cell wall, are very digestible. Thus, they have gained more attention in recent years as a food supplement, especially for athletes and vegetarians [2].

However, the undesirable taste, low digestibility, and high allergenicity of algae-based protein isolates for monogastric animals and humans have limited their application in the food industry. Protein hydrolysates and peptides can be excellent alternatives to overcome the problems associated with the direct consumption of protein isolates, as they have nutritional and health-promoting features and act as natural antioxidant agents in food preservation [3,4]. Although algae-derived peptides have various advantages, their instability during storage and under harsh conditions (e.g., in the gastrointestinal tract), low absorption efficiency, bitterness, reaction with the food matrix, and possible inactivation inhibit the application of hydrolysates in foods and beverages. Incorporating these bioactive compounds in lipid-based nanocarriers such as liposomes can be an appropriate solution to cover all of these problems and increase their efficacy under different conditions [5].

Liposomes have an enclosed vesicular structure and are able to accommodate both water-soluble and hydrophobic compounds in their internal aqueous core and bilayer space, respectively [6]. Moreover, the typical constituents of liposomes are completely natural, and their nontoxicity has led to the broad application of these vesicular systems in the encapsulation of various bioactive compounds [7,8]. However, the major drawbacks of this versatile carrier are the fluidity of the intravesicular space and the flexible bilayer structure, which can lead to the physical instability of vesicles (aggregation/flocculation and fusion/coalescence), resulting in changes in size and loss of liposome-incorporated bioactive materials over time. A possible solution to this problem is to engineer the liposomal structure [9]. Mostly, cholesterol has been applied as a stabilizing factor in vesicular systems because it can increase the packing of phospholipid molecules, reduce the fluidity of intravesicular space, and consequently create a more rigid and stable structure over time and under severe shear stress [10]. Moreover, the above-mentioned problems can be improved by depositing an oppositely charged biopolymer such as chitosan around the liposome surface through electrostatic interaction [11,12]. Several studies on improving hydrolysate stability during storage and processing using chitosan coating have been conducted [13,14].

To make a formulation that is more stable over time and more appropriate for industrial application, it can be transformed into powder form by spray-drying or freeze-drying. Freeze-drying is a more expensive technology and requires more time and energy compared with spray-drying. Thus, spray-drying technology is an economical strategy to make powdered liposomal dispersions, and it has a wide range of use in the food industry compared to other drying techniques. However, there have been no studies regarding the simultaneous use of different stabilizers and coating materials to increase the stability and bioactivity of liposome-containing *Spirulina* hydrolysates. Therefore, the aims of this research were as follows: (1) to explore the effect of cholesterol and γ-oryzanol as stabilizing agents and chitosan as a coating material on the mean diameter and encapsulation efficiency of *Spirulina* hydrolysate-loaded liposome, (2) to examine the antioxidant capacity of synthesized liposomes using the different methods, and (3) to estimate the stability of synthesized liposomes during storage. Finally, the optimum formulation for easy usage was converted into powder form and its physicochemical and structural properties and antioxidant activity were evaluated.

## 2. Materials and Methods

### 2.1. Materials and Reagents

Pepsin from powdered porcine gastric mucosa (activity ≥ 250 units/mg solid), 1,1-diphenyl-2-picrylhydrazyl (DPPH), 2,2′-azino-bis(3-ethylbenzothiazoline-6-sulfonic acid) diammonium salt (ABTS), 2,4,6(tripyridyl)-1,3,5-triazine (TPTZ), cholesterol, and Coomassie brilliant blue (G250) were purchased from Sigma-Aldrich (St. Louis, MO, USA); potassium persulfate, iron (III) chloride hexahydrate, trichloroacetic acid (TCA), ferrous chloride, γ-oryzanol, iron sulfate, and maltodextrin were obtained from Merck (Darmstadt, Germany). *Spirulina platensis* powder was purchased from Noor Daro Gonbad (Gonbad Kavous, Iran).

### 2.2. Protein Hydrolysis

For protein hydrolysis, lyophilized *Spirulina platensis* protein was dispersed in distilled water (DW) to achieve a protein concentration of 3% (*w*/*v*). Protein hydrolysates were produced by pepsin protease for 240 min. The hydrolysis conditions were set as follows: pH 2, temperature 37 °C, enzyme-to-substrate (E/S) ratio of 6% (*w/w*). The hydrolysis reaction was performed in a shaker incubator (Unimax 1010; Heidolph, Schwabach, Germany), then the enzymes were thermally inactivated (90 °C, 10 min), and the solution was cooled down to room temperature. The hydrolysate solution was then centrifugated at 4550× *g* for 10 min and the supernatant was collected for further analysis [4].

### 2.3. Degree of Hydrolysis (DH)

The extent of enzymatic hydrolysis can be defined by the degree of hydrolysis (DH), which is a key factor determining the chain length of peptides, and thereby their functional properties. A higher DH corresponds to mean shorter peptide length and vice versa. This index is significantly influenced by hydrolysis time and the type of enzyme used. To determine DH, 1 mL of hydrolysate was added to 1 mL of TCA (0.44 M), followed by centrifugation at 7800× *g* for 10 min at 4 °C. The collected supernatants were analyzed for soluble protein by the Bradford assay. DH was estimated using the following equation [15]:

(1) $$\mathrm{DH}\;(\%)=\frac{\mathrm{TCA}\;-\;\mathrm{Soluble}\;\mathrm{Protein}\;\mathrm{of}\;\mathrm{Hydrolysate}}{\mathrm{Total}\;\mathrm{Protein}\;\mathrm{of}\;\mathrm{Sample}\;(\mathrm{non}-\mathrm{hydrolyzed})}\times 100$$

### 2.4. Amino Acid Profile

An RP-HPLC apparatus (Young Lin Acme 9000, YL Instruments, Anyang, Korea) equipped with a reverse-phase column (150 mm × 4.6 mm × 5 μm; RP-C18 ODS-A, Barcelona, Spain), a fluorescence detector (LC305; Lab Alliance, State College, PA, USA), and a mobile phase of acetate buffer (50 mM at pH 3.4, with a flow rate of 1.3 mL/min) were used to determine the amino acids in *Spirulina* protein hydrolysates. For this purpose, the hydrolysate was intensively treated with HCl (6 M) at 110 °C for 24 h. The digested sample was derivatized with orthophthaldehyde and injected to the HPLC column. The amount of amino acids in hydrolysates was expressed as mg/100 g protein. The biological value (*BV*) and amino acid score (*AAS*) of hydrolysates, as nutritional parameters, were determined using the following equations [16]:

(2) $$AAS=\frac{\%\;\mathrm{Essential}\;\mathrm{amino}\;\mathrm{acids}\;\mathrm{in}\;\mathrm{sample}}{\%\;\mathrm{Essential}\;\mathrm{amino}\;\mathrm{acids}\;\mathrm{recommended}\;\mathrm{by}\;\mathrm{FAO}}$$

(3) $$BV\;\left(\%\right)=10^{2.15}\times Lys^{0.41}\times {\left(Phe+Tyr\right)}^{0.6}\times {\left(Met+Cys\right)}^{0.77}\times Thr^{0.24}\times Trp^{0.21}$$

where each amino acid symbol is expressed as % amino acid in sample/% amino acid FAO pattern.

### 2.5. Preparation of Spirulina Hydrolysate (HS)-Loaded Liposomes

HS-encapsulated nanoliposomes were prepared using the thin layer hydration method as described by Mohammadi et al. [7] with slight modifications. For this procedure, 1.2 % (*w*/*v*) Phospholipon 90 G (soybean lecithin of ~90% phosphatidylcholine; Lipoid GmbH, Ludwigshafen, Germany) and 2 stabilizing agents (cholesterol or 0.15% (*w*/*v*) γ-oryzanol) were dissolved in 15 mL of 96% ethanol and stirred on a hotplate at 50 °C for complete solubilization. Subsequently, the solvent was evaporated using a rotary evaporator (Heidolph, Germany) at 50 °C until a thin film was formed in the round-bottomed flasks. The resulting lipid films were hydrated with 15 mL of DW containing HS at 0.3% (*w*/*v*) with continuous agitation on a rotary evaporator at 55 °C, followed by sonication using a sonication probe (130 W, 20 kHz; Vibra-Cell Sonics & Materials, Newtown, CT, USA) at 80% sonication strength for 10 min. During sonication, the sample was placed into an ice bath to avoid overheating of dispersion. To prepare the empty liposomes, the same method was applied, except HS was excluded in the hydration step and the thin layer was hydrated only with DW.

### 2.6. Preparation of Chitosan-Coated Nanoliposomes

For coating with chitosan, prepared nanoliposomes were added to chitosan solution (0.4%, *w*/*v*) dissolved in acetic acid (1% *v/v*) in a drop-wise manner with a volume ratio of 1:1 and stirred for 2 h.

### 2.7. Characterization of HS-Loaded Liposomes

#### 2.7.1. Measurement of Particle Size and Zeta Potential (ζ)

Liposome dispersions were diluted 1:10 with DW before analysis by a zetasizer (Zetasizer Nano ZS, Malvern Instruments Ltd., Malvern, UK).

#### 2.7.2. Encapsulation Efficiency

Encapsulation efficiency (*EE*) was determined by separating encapsulated hydrolysates from free ones using an Amicon filter (Amicon Ultra-15, with molecular weight cutoff of 30 kDa; Millipore Corp., Cork, Ireland), followed by centrifugation at 3000 rpm for 10 min. Free and total hydrolysates were determined by calculating the protein amount using the Bradford method as described previously.

*EE* was determined according to following equation:

(4) $$EE=\frac{\;\mathrm{Total}\;\mathrm{protein}\;\mathrm{content}-\mathrm{Amount}\;\mathrm{of}\;\mathrm{free}\;\mathrm{hydrolysate}\;}{\mathrm{Total}\;\mathrm{protein}\;\mathrm{content}}\times 100$$

#### 2.7.3. Scanning Electron Microscopy (SEM)

To investigate the morphological features of the vesicles, γ-oryzanol liposome (with and without chitosan coating) was dispersed onto the laboratory lamel and dried at 37 °C, then transferred to adhesive-coated aluminum pin stubs. The stubs were coated with a thin layer of gold and examined using a scanning electron microscope (MIRA3, TESCAN, Brno, Czech Republic) [17].

#### 2.7.4. Transmission Electron Microscopy (TEM) Measurements

For TEM measurement, 5 µL of each sample was placed onto a copper grid coated with carbon film for 3 min before being blotted off using filter paper. After that, 10 µL of contrast dye containing 2% uranyl acetate was placed onto the grid, left for 2 min, and blotted off with filter paper. Finally, the grids were loaded onto a specimen holder and then into a transmission electron microscope (100 Kv; LEO 906, Zeiss, Oberkochen, Germany).

#### 2.7.5. Fourier-Transform Infrared Spectroscopy (FT-IR)

To determine the functional groups of liposomes, lyophilized samples (ALPHA 1–4 LD freeze dryer, Martin Christ, Osterode am Harz, Germany) were formed into KBr pellets with a mass ratio of 1:100 [17]. The samples were analyzed using FTIR (4300, Shimadzu, Kyoto, Japan) from 4000 to 400 cm^−1^ with a minimum of 256 scans/spectrum and a constant scan speed of 4°/s.

#### 2.7.6. Determination of Total Phenolic Content (TPC)

TPC was performed according to the Folin–Ciocalteu method described by de Araujo et al. [18]. Briefly, 300 µL of sample was mixed with 125 µL of Folin–Ciocalteu reagent and 1825 µL of DW. After the mixture was vortexed for 5 min at ambient temperature, 250 µL of sodium carbonate solution (20%, *w*/*v*) was added and it was vortexed for another 5 min. Then, the mixture was placed in a water bath at 40 °C for 30 min. The samples were then centrifuged at 10,000 rpm for 10 min, and the absorbance of the upper phase was measured at 765 nm by a spectrophotometer (Ultrospec 2000; Scinteck, Cambridge, UK). The results were expressed as mg gallic acid per g sample using the following formula [18]:

(5) $$C=c\frac{V}{m}$$

where *C* is the total phenolic content (mg GAE/g dry extract), *c* is the concentration of gallic acid obtained from the calibration curve (mg/mL), *V* is the volume of extract (mL), and m is the mass of the extract (g).

#### 2.7.7. Antioxidant Activity of Protein Hydrolysates

##### 2.7.7.1. DPPH Radical Scavenging Activity

To measure DPPH radical scavenging activity, 1 mL of each concentration of hydrolysates was added to 1 mL of DPPH solution (0.1 mM), followed by incubation for 30 min in the dark. The absorbance was read at 517 nm and the DPPH radical scavenging activity was calculated by the following equation [19]:

(6) $$\mathrm{DPPH}\;radical\;scavenging\;activity\;\left(\%\right)==\frac{A_{control}-A_{sample}}{A_{control}}\times 100$$

where *A_control_* and *A_sample_* are the absorbance of the control and sample, respectively.

##### 2.7.7.2. ABTS Radical Scavenging Activity

The mixture of ABTS (7 mM) and potassium persulfate (2.45 mM) with a volume ratio of 1:1 generates a green-blue reagent (ABTS^+^) after 12–16 h incubation in the dark, and has maximum absorption at 734 nm. When this cationic radical is exposed to the hydrogen donating compound, the green-blue is decolorized and the color intensity is measured at 734 nm. To measure ABTS radical scavenging activity, 40 μL of the prepared hydrolysate concentration was mixed with 4 mL of diluted ABTS solution, vortexed vigorously for 30 s, and incubated in the dark for 6 min. The absorbance was measured at 734 nm. The ABTS radical scavenging activity was calculated by the following equation [20]:

(7) $$\mathrm{ABTS}\;radical\;scavenging\;activity\;\left(\%\right)=\frac{A_{control}-A_{sample}}{A_{control}}\times 100$$

##### 2.7.7.3. Ferric Reducing/Antioxidant Power (FRAP) Assay

The capability of hydrolysate to reduce Fe^+3^ ions present in the complex to a Fe^+2^ form with 2,4,6-tri (2-pyridyl)-s-triazine (TPTZ) was determined by the ferric ion reducing capacity (FRAP) assay as described previously. The FRAP reagent was freshly prepared by mixing TPTZ (10 mM) dissolved in 40 mM HCL, iron (III) chloride hexahydrate (20 mM) dissolved in water, and acetate buffer (0.3 mM) at pH 3.6 at a ratio of 1:1:10 (*v/v/v*) and warming it to 37 °C. Then, 900 μL of the working solution was mixed with 100 μL of different concentrations of hydrolysate and the corresponding nano-formulated system, followed by incubation of the mixture at 37 °C for 30 min, and the resulting blue color absorbance at 595 nm was recorded. Different concentrations of FeSO_4_, in the range 0–1 mM, were used as the calibration curve [21].

### 2.8. Storage Stability of HS-Loaded Liposome and Chitosome

In order to perform this test, HS-loaded liposomes (stabilized with γ-oryzanol) and the corresponding chitosomes were stored at 4 °C for 1 month. The mean diameter, PDI, ζ-potential, and precipitation were monitored during storage by DLS as described in Section 2.7. The residual antioxidant activity of selected formulations was controlled by ABTS assay.

### 2.9. Spray-Drying of Chitosomes

Before spray drying, the γ-oryzanol-stabilized chitosome dispersion was mixed with maltodextrin solution (40% *w*/*v*) at a mass ratio of 40:60, and stirred overnight at room temperature. Then, the resulting dispersions were spray-dried using a Mini Spray Dryer (Büchi Labortechnik, Flawil, Switzerland). The inlet and outlet air temperature were 130 and 75 °C, respectively. Dried powders were stored in airtight containers and placed in a desiccator at room temperature.

### 2.10. Characterization of Spray-Dried Powder

Production yield was measured by calculating the mass ratio of the produced powder to the total solid content in the feed. Other physical properties of the spray-dried powder, such as moisture content, bulk density, and solubility, were computed using the methods described by Sarabandi et al. [20].

The particle morphology of spray-dried powder was evaluated by scanning electron microscopy (SEM; MIRA3, TESCAN, Brno, Czech Republic).

To determine the size, polydispersity, and ζ of reconstituted nanoliposomes, the powder was dissolved in an appropriate concentration and its particle size and ζ potential were determined by a zetasizer.

### 2.11. Statistical Analysis

Statistical analysis was performed using SPSS software (version 24.0, IBM, Chicago, IL, USA). Normal distribution and variance homogeneity had been previously tested (Shapiro–Wilk). Data of 3 repetitions were subjected to analysis of variance (ANOVA), followed by Tukey’s test at a 5% significance level.

## 3. Results and Discussion

### 3.1. Characterization of Hydrolyzed Spirulina Protein (HS)

Extracted *Spirulina* isolate was hydrolyzed by pepsin enzyme. The hydrolysis degree was found to be 16.5% over 4 h hydrolysis time. The solubility of hydrolysate under harsh acidic conditions was improved after enzymatic hydrolysis, but the highest solubility was obtained under alkaline pH conditions.

**Figure 1:**
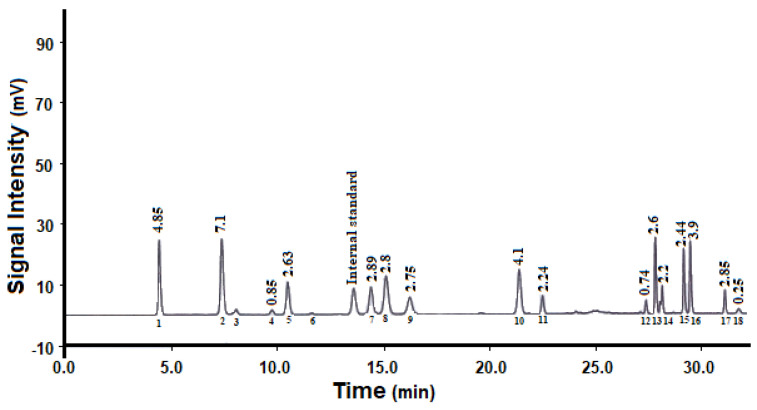
Amino acid composition of pepsin-hydrolyzed peptides: (1) aspartic acid; (2) glutamic acid; (3) asparagine; (4) histidine; (5) serine; (6) glutamine; (7) arginine; (8) glycine; (9) threonine; (10) alanine; (11) tyrosine; (12) methionine; (13) valine; (14) phenylalanine; (15) isoleucine; (16) leucine; (17) lysine; (18) tryptophan.

Figure 1 shows the amino acid composition of pepsin-hydrolyzed peptides. According to the obtained profile, the hydrolysate was rich in acidic amino acids (aspartic and glutamic acid), arginine, valine, lysine, alanine, glycine, threonine, and leucine. All the essential amino acids (except sulfur-containing amino acids) were present in the hydrolysate at concentrations higher than the FAO recommended levels for adults. Moreover, the hydrolysate showed good nutritional value as determined by amino acid score (72%) and biological value (78%), and good antioxidant activity (IC_50_ 1 mg/mL).

Compared to native protein (with a DPPH IC_50_ value of 3 mg/mL), the hydrolysates had a significantly lower IC_50_ value of 1.0 mg/mL, indicating their effectiveness in scavenging DPPH radicals. The ABTS IC_50_ of hydrolysate was estimated to be 2 mg/mL. Compared with native protein (with an IC_50_ value of 4.5 mg/mL), the hydrolysates had lower IC_50_ values, indicating their effectiveness against ABTS radicals.

Encapsulating these bioactive compounds in lipid-based nanocarriers such as liposomes can be an appropriate solution to cover all of the mentioned problems and increase their efficacy under different conditions.

### 3.2. Characterization of Uncoated and Chitosan-Coated Liposomal Dispersions

In this study, HS was encapsulated into the liposomal carrier and its experimental characteristics were investigated. Two stabilizers (cholesterol and γ-oryzanol) were applied in the preparation of primary liposome dispersion, then the resulting nanoliposomes were coated with cationic chitosan polymer at a final concentration of 0.2% *w*/*v* (this concentration resulted in the smallest particle size and highest surface charge on the coated liposome dispersions), and their effects on the physicochemical properties of the resulting formulations were examined. The mean particle diameter and ζ of primary and chitosan-coated nanoliposomes (chitosomes) are shown in Table 1. Liposomes stabilized by cholesterol and γ-oryzanol had a small particle size and homogeneous size distribution, and their surface charge was between −11 and −14 mV. Following the addition of the chitosan polymer, the particle size and PDI of nanoliposomes increased and ζ changed from negative to positive values (approximately 29 mV), confirming that cationic chitosan successfully covered the primary liposomes. These findings are in accordance with those of Altin et al. [22], who reported that surface coating of primary liposomes containing phenolic extract from cocoa hull waste with cationic chitosan by electrostatic deposition increased the particle size of liposomes and the secondary liposomes had a positive charge.

**Figure 2:**
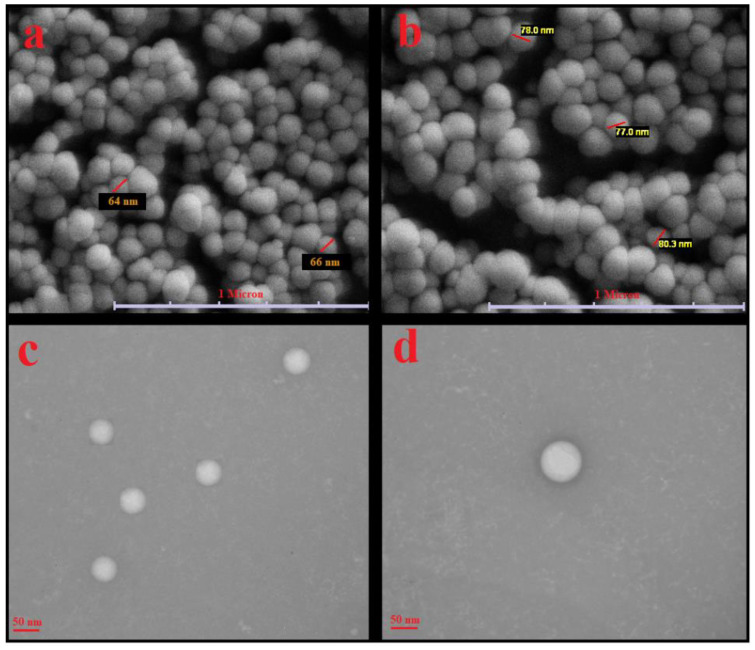
(**a**,**b**) SEM and (**c**,**d**) TEM images of primary liposomes stabilized with γ-oryzanol (**a**,**c**) and corresponding γ-oryzanol-chitosomes (**b**,**d**).

SEM and TEM images of primary liposomes (γ-oryzanol-liposomes) and the corresponding chitosomes are shown in Figure 2. The cholesterol-liposome and γ-oryzanol-liposome had similar shape and morphology. The results obtained from the zetasizer apparatus were somewhat confirmed by SEM and TEM. The SEM images show spherical particles with a small particle size < 100 nm and narrow distribution. In TEM images, the spherical structure and monodispersed distribution of primary liposomes are very clear [23].

### 3.3. Encapsulation Efficiency (EE)

The *EE* of the primary HS-loaded liposomes stabilized by cholesterol and γ-oryzanol is given in Table 1. Overall, both liposomes showed high *EE* > 85%, indicating that both stabilizing agents had good potential for encapsulation of HS. Incorporating sterol compounds in the liposome structure significantly increased the rigidity of the liposome membrane; thus, the system could encapsulate a larger amount of hydrophilic bioactive material. In another study, high *EE* was reported for fish hydrolyzed collagen-loaded liposomes stabilized by cholesterol and glycerol [24]. In another study, orange seed protein hydrolysates were produced using alcalase and pepsin enzymes, which were incorporated into uncoated liposomes and chitosome systems. The hydrolysates produced with alcalase showed a higher *EE* than those produced with pepsin. The authors suggested that this difference may be related to the higher DH of alcalase hydrolysate (approximately 24%), the lower molecular weight of resulting peptides compared to pepsin hydrolysate, and the easy incorporation of alcalase hydrolysate into the aqueous core of liposomes [14]. The authors also claimed that incorporating peptides into the chitosomes led to increased *EE* of vesicles compared to plain liposomes. This may be related to occupying pores in the surface of the liposome surface preventing the leakage of incorporated bioactive materials. These findings were consistent with those reported by [14,20].

### 3.4. Determination of Total Phenolic Content and Antioxidant Activity of Liposomes and Chitosomes

Liposomal nanocarriers can be applied for encapsulation of both liposoluble and hydrophilic antioxidant and phenolic compounds to improve their bioavailability. The total phenolic and antioxidant capacity of uncoated liposomes and chitosome dispersions are shown in Table 2. There was no significant difference (*p* < 0.05) between HS and HS-loaded cholesterol-liposomes by TPC, DPPH, ABTS, or FRAP assay, indicating that the phenolic compounds and, subsequently, the antioxidant properties of HS were properly preserved in the nanoliposomal carrier. Preservation of the antioxidant activity of anthocyanin-rich black carrot extract after 21 days of storage by encapsulating in liposomes has been reported [25].

After the cholesterol-liposome surface was coated with chitosan, the antioxidant activity remained unchanged as compared to the uncoated liposome. The antioxidant activity of chitosan has been reported by others [22]. The authors suggested that the phenolic bioactive material could be partially located on the surface of the liposome, and consequently chitosan–phenolic compound conjugates might be formed, and these couples synergistically improve the antioxidant activity [22].

Conversely, in another study, after coating the surface of sour cherry extract-loaded liposomes with cationic chitosan, the TPC content decreased from 38.19 to 31.23 mg/L. The authors suggested that the available chitosan on the liposome surface might block the availability of phenolic compounds on the surface of uncoated liposomes [26].

γ-Oryzanol, a plant sterol with a structure similar to cholesterol, has a wide capacity for scavenging free radicals, consequently preventing lipid oxidation. The HS-loaded γ-oryzanol-liposomes showed higher TPC and antioxidant capacity. This was attributed to the cooperative scavenging capacity of γ-oryzanol with HS in a liposome system [27]. This cooperative antioxidative effect was reported by Li et al. [28]. Sage extract (SE) and zein hydrolysate in combination showed higher antioxidant activity than the simple sum of their individual effects [28].

### 3.5. Fourier-Transform Infrared Spectroscopy (FTIR)

**Figure 3:**
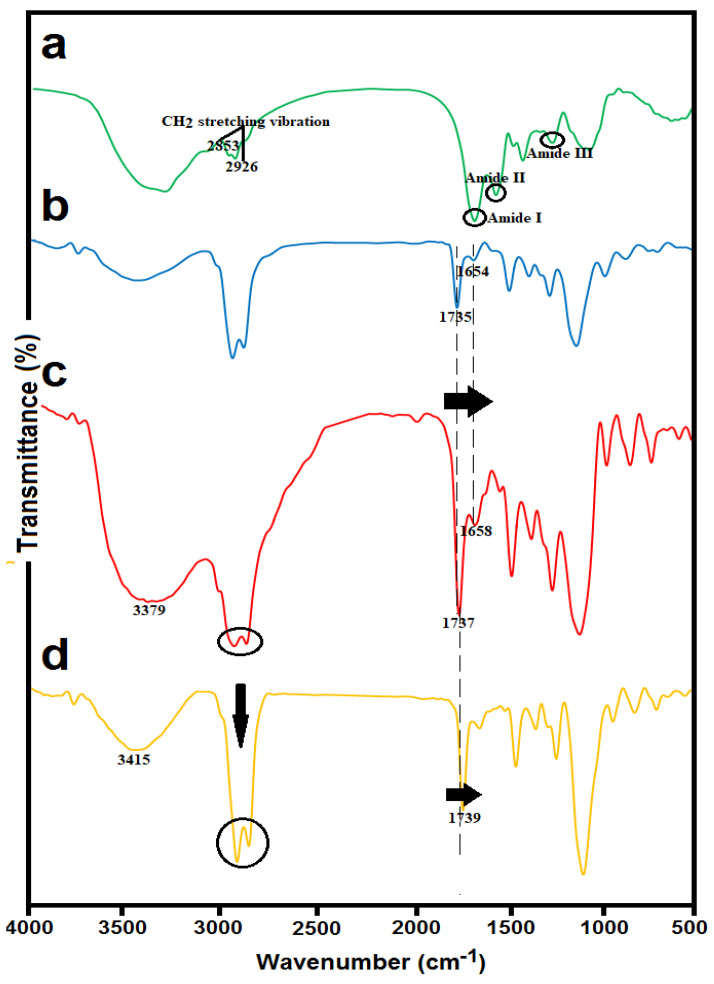
FTIR spectra of (**a**) *Spirulina* hydrolysate (HS); (**b**) blank nanoliposomes stabilized with γ-oryzanol; (**c**) HS-loaded γ-oryzanol nanoliposomes; and (**d**) HS-loaded γ-oryzanol-chitosomes.

The structural changes in synthesized liposomes with and without HS and the successful chitosan coating were confirmed by FTIR spectroscopy. From the IR spectrum of the HS (Figure 3a), a broadband at 3300 cm^−1^ was attributed to O–H and N–H stretching and two bands at 2926 and 2853 cm^−1^ were related to CH_2_ stretching vibrations of aliphatic chains. The amide region bands (1658 cm^−1^ corresponding to protein amide I, 1550 cm^−1^ corresponding to protein amide II, and 1247 cm^−1^ related to protein amide III) were clearly visible in the IR spectrum of the HS [29].

Blank nanoliposomes (Figure 3b) were observed at the following wavenumbers: 3438 cm^−1^ related to hydroxyl stretch vibration, 2925 cm^−1^ attributed to stretch vibrations of a methylene group, 1735 cm^−1^ mainly related to the stretching vibration of the polar head ester groups of phospholipids, 1654 cm^−1^ related to C=C stretching vibrations, 1246 and 1109 cm^−1^ corresponding to symmetric and antisymmetric stretch vibrations of a phosphate group, and 958 cm^−1^ related to asymmetrical stretch vibrations of N^+^/CH_3_.

Comparing the IR spectra of HS-loaded γ-oryzanol-liposomes (Figure 3c) and corresponding blank liposomes (Figure 3b), a great similarity between their spectra can be observed. The incorporation of hydrolysate into the liposomal carrier resulted in a shift in some frequencies. The most important of these changes were slight shifts at 1735 and 1654 cm^−1^ to 1737 and 1658 cm^−1^ for HS-loaded liposomes, which may correspond to the possible interaction of HS with carbonyl ester groups at the interfacial part of the liposomal bilayers [30].

For the HS-loaded γ-oryzanol-chitosomes (Figure 3d), a shift from 3379 to 3415 cm^−1^ was detected, which may correspond to hydrogen bonding between hydroxyl or amino groups of chitosan and carboxylic acid or amino groups of hydrolysates. Moreover, after chitosan coating, significant changes in the absorption bands of acyl chains (3000–2800 cm^–1^) were detected. These peaks were converted into two narrow and intense peaks at 2862 and 2924 cm^−1^ in the case of chitosan-coated liposomes. Further evidence for electrostatic conjugation of chitosan on liposome surface was a considerable shift to higher frequencies in the carbonyl group (from 1737 to 1739 cm^–1^). This indicates that the carbonyl groups are involved with cationic groups of chitosan, resulting in the destruction of some hydrogen bonds [31].

### 3.6. Storage Stability of HS-Loaded Liposomes and Chitosomes

Regarding our previous research on the effect of temperature on the physical stability of vitamin D_3_-loaded liposomes and the instability and aggregation of the formulation at ambient temperature due to higher fluidity of the lipid bilayer and higher loss of encapsulated bioactive material, refrigerator temperature (4 °C) was selected to examine the physical stability of selected formulations [7]. Liposomes as vesicular droplets have permeable and flexible bilayers and a high tendency to fuse and aggregate, resulting in the release of encapsulated bioactive materials during storage. In this study, stabilizing agents (cholesterol and γ-oryzanol) and coating material (chitosan) were tested to enhance the physical stability of liposomes. HS-loaded γ-oryzanol-liposomes and the corresponding chitosomes had no marked difference (*p* < 0.05) in magnitude, PDI, and ζ after 30 days of storage at 4 °C (Table 3). Moreover, both samples preserved more than 90% of their initial antioxidant activity in ABTS radical scavenging activity (*p* < 0.05). When the storage time was expanded, slight lipid oxidation may have occurred in unsaturated fatty acids of the phospholipid bilayers, leading to decreased antioxidative activity of the tested formulations. On the other hand, no significant precipitate was observed, especially in the HS-loaded chitosome system, during storage. When storage time was expanded to 2 months, the chitosome system showed higher physical stability compared to the uncoated liposomes. In contrast, the stability of HS-loaded cholesterol-liposomes in terms of mean diameter, PDI, and ζ was much lower than that of HS-loaded γ-oryzanol-liposomes at 4 °C. When storage time was expanded, the mean diameter and PDI of these formulations significantly increased (*p* < 0.05), and observable precipitates were separated into round-bottomed Falcon tubes.

On the other hand, the residual antioxidant activity of HS-loaded cholesterol-liposomes was 50% of its initial antioxidant activity. This observed instability of HS-loaded cholesterol-liposomes could have resulted from the poor coating of liposomal space cores with cholesterol stabilizing agents, and possible replacement of empty spaces with hydrophilic domain residues (glycine, arginine, and lysine) of hydrolysates. Nevertheless, some hydrophobic domains of *Spirulina* hydrolysates could interact with acyl chains of the lipid bilayer of liposome through hydrophobic binding, leading to more flexibility and fluidity of liposomes [32]. HS-loaded γ-oryzanol-chitosomes showed better results in terms of particle size, PDI, aggregation, and antioxidant activity compared to HS-loaded cholesterol-liposomes during storage. In summary, HS-loaded γ-oryzanol-liposomes and HS-loaded γ-oryzanol-chitosomes showed promising storage stability with no precipitate or change in mean diameter and a slight decrease in antioxidant activity at 4 °C. Thus, γ-oryzanol as a stabilizing agent and chitosan polymer as a coating material were able to reduce membrane fluidity and flexibility, contributing to stability.

In another study, different stabilizers (cholesterol and glycerol) were used to encapsulate peptides obtained from defatted Asian sea bass skin. Regarding the results, both formulations showed small particle size and high encapsulation efficiency. However, after the lyophilization process, hydrolysate-loaded cholesterol-liposomes showed higher stability and higher antioxidant activity in the gastrointestinal tract than lyophilized glycine-liposomes during storage at 25 °C for 28 days [24]. It was reported that curcumin-loaded chitosomes showed higher physical stability than curcumin-loaded uncoated liposomes due to the lower flexibility of chitosan-coated liposomes and, as a result, lower membrane fusion between droplets [33].

### 3.7. Production Yield and Physicochemical Properties of Spray-Dried Chitosomes

The powder moisture content was found to be 4.65 ± 0.61%, which is lower than the specified minimum moisture content of many powders used in food applications to inhibit microbiological spoilage and lipid oxidation and extend the product’s shelf life.

The bulk density of the powder was 0.31 ± 0.01 g cm^−3^. A higher bulk density has several advantages, including more convenient storage conditions due to lower space requirements for storage and greater protection against oxidation during storage due to the presence of less air in powder. In addition, the solubility and production yield of powder were found to be 96% and 57%, respectively.

### 3.8. Microstructure and Particle Size Distribution

**Figure 4:**
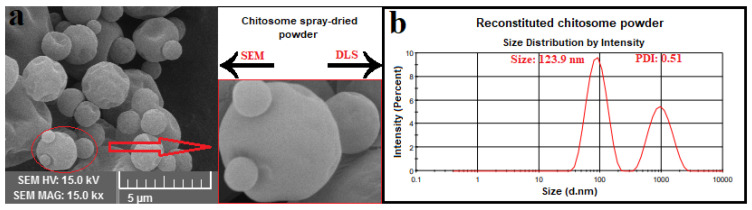
(**a**) SEM image of spray-dried hydrolysate-loaded γ-oryzanol-chitosomes; (**b**) particle size result of reconstituted γ-oryzanol-chitosome powder.

The morphology of dried particles is mostly affected by the evaporation rate and viscoelastic properties of shell material. SEM images (Figure 4) show mostly spherical particles with little evidence of roughness or fracturing in the microcapsules. Moreover, most of the spray-dried powders showed a well-defined spherical shape with a particle size of around 1–3 μm, which is smaller than the threshold diameter used in food fortification (10–50 μm) [34]. The coarse powder results in a sandy and unfavorable mouthfeel in the fortified food products. These results are in accordance with those reported by Sarabandi et al. [13].

### 3.9. Properties of Reconstituted Chitosomes

#### 3.9.1. Physical Properties

It is essential to conserve the physicochemical properties of nanoliposomes after the spray-drying process. The effect of spray-drying on particle size, PDI, and ζ of reconstituted chitosomes was investigated (Table 4). As expected, the chitosan coating increased the physical stability of nanocarriers after spray-drying, and slight changes were observed in the size and ζ of reconstituted chitosomes, but the PDI for these systems changed from 0.33 to 0.51. In a previously reported study, the effect of spray-drying on the physical properties of reconstituted liposomal powders was investigated. Regarding those results, the uncoated liposome systems were immediately unstable, as the maltodextrin was added to the dispersion as a wall matrix, i.e., these systems were not spray-dried. However, the coated systems did not significantly affect the spray-drying process and the diameter of the liposomal powder was smaller than that of liposomal dispersion before thermal processing. The authors suggested that when coated liposomal systems are added to a solution containing salts or sugars, which have the potential to induce an osmotic driving force and reduce water activity, migration of water molecules happens from the core to the liposomal surface, reducing the concentration gradient between the internal and external aqueous phases of the liposomes, thus reducing the size of the liposomal powder [35]. Moreover, the addition of maltodextrin as a hydrophilic nonionic polysaccharide had no effect on the ζ of coated liposomes.

#### 3.9.2. Retention of Antioxidant Activity (AA)

The effect of spray-drying and thermal stress on the retention of ABTS radical scavenging activity in the chitosome system is shown in Table 4. There was no significant difference between these two indices in chitosome system retention before and after the spray-drying process (*p* < 0.05), which shows the positive effect of chitosan coating on the preservation of the biological activity of peptide fractions against thermal stresses during spray-drying. Our results are consistent with those reported by Sarabandi et al. [13]. In another study, about 39% of total phenols, 30% of flavonoids, and 47% of radical scavenging activity of extract were maintained after the spray-drying of nanoliposomes [22]. The spray-drying of nanoliposomes loaded with black mulberry extract resulted in the preservation of approximately 69% of total phenolic and 56% of anthocyanin compounds in chitosan-coated nanoliposomes [36].

## 4. Conclusions

*Spirulina plantensis* hydrolysate was successfully encapsulated into nanoliposomes using the thin-layer hydration method of sonication. This study shows that stabilizing the liposome structure with γ-oryzanol and covering the liposomes with a polycationic chitosan polymer provided long-term physical stability, and the system preserved its antioxidant capacity over time. Thus, this study suggests that chitosomes stabilized with γ-oryzanol could be used as a promising delivery system to protect against the loss of hydrolysate under processing or storage conditions.

## Figures and Tables

**Table 1:** Characteristics of hydrolysate-loaded γ-oryzanol-liposome and chitosome.

	Z-Average (nm)	PDI	ζ (mV)	Encapsulation Efficiency (%)
Cholesterol-liposome	56.6 ± 2.1 ^c^	0.17 ± 0.02 ^d^	−11.1 ± 1.5 ^b^	85.0 ± 1.2 ^a^
γ-Oryzanol-liposome	63.9 ± 1.8 ^b^	0.18 ± 0.02 ^c^	−14.8 ± 1.1 ^b^	87.0 ± 1.3 ^a^
Cholesterol-chitosome	66.8 ± 2.4 ^b^	0.27 ± 0.03 ^b^	24.8 ± 1.2 ^a^	88.0 ± 1.0 ^a^
γ-Oryzanol-chitosome	78.4 ± 1.9 ^a^	0.28 ± 0.01 ^a^	26.9 ± 1.5 ^a^	89.0 ± 2.0 ^a^

Means in same column with different superscripts (a, b, c, d) are statistically different (*p* < 0.05).

**Table 2:** Antioxidant activity and total phenol content of hydrolysate-loaded γ-oryzanol-liposomes and chitosomes.

	Total Phenol (mg Gallic Acid/g Extract)	FRAP Assay (µM FeSO_4_/mL)	Inhibition of ABTS Radical (%)	Inhibition of DPPH Radical (%)
Pepsin hydrolysate	51.0 ± 1.4 ^b^	400.0 ± 3.1 ^b^	53.0 ± 1.2 ^b^	50.0 ± 1.5 ^b^
Cholesterol-liposome	53.6 ± 1.6 ^b^	395.0 ± 3.5 ^b^	52.0 ± 2.1 ^b^	47.0 ± 2.0 ^b^
γ-Oryzanol-liposome	152.9 ± 2.1 ^a^	650.0 ± 2.8 ^a^	85.0 ± 2.5 ^a^	90.0 ± 1.8 ^a^
Cholesterol-chitosome	55.6 ± 2.4 ^b^	410.0 ± 3.4 ^b^	54.0 ± 2.1 ^b^	48.0 + 2.1 ^b^
γ-Oryzanol-chitosome	155.0 ± 1.9 ^a^	655.0 ± 3.1 ^a^	86.0 ± 1.9 ^a^	88.0 ± 2.3 ^a^

Means in same column with different superscripts (a, b) are statistically different (*p* < 0.05).

**Table 3:** Physical stability of hydrolysate-loaded γ-oryzanol-liposomes and chitosomes during one month at 4 °C.

	Z-Average (nm)	PDI	ζ (mV)	Antioxidant Activity (ABTS Assay)
Pepsin hydrolysate	-	-	-	25.0 ± 1.8 ^b^
Cholesterol-liposome	350.6 ± 2.1 ^a^	0.31 ± 0.02 ^b^	−9.1 ± 1.5 ^b^	28.0 ± 2.1 ^b^
γ-Oryzanol-liposome	90.9 ± 1.8 ^d^	0.19 ± 0.02 ^d^	−13.8± 1.1 ^b^	75.0 ± 2.5 ^a^
Cholesterol-chitosome	220.8 ± 2.4 ^b^	0.38 ± 0.03 ^a^	20.8 ± 1.2 ^a^	38.0 ± 2.1 ^b^
γ-Oryzanol-chitosome	120.4 ± 1.9 ^c^	0.29 ± 0.01 ^c^	24.9± 1.5 ^a^	76.0 ± 1.9 ^a^

Means in same column with different superscripts (a, b, c, d) are statistically different (*p* < 0.05).

**Table 4:** Physicochemical properties of reconstituted hydrolysate-loaded γ-oryzanol-chitosomes.

	Z-Average (nm)	PDI	ζ (mV)	Residual Antioxidant Activity (%)
HS-loaded chitosome dispersion	96.9 ± 1.8 ^b^	0.33 ± 0.02 ^b^	31.4 ± 1.5 ^a^	86.0 ± 1.2 ^a^
HS-loaded chitosome powder	123.9± 2.1 ^a^	0.51 ± 0.02 ^a^	33.2 ± 1.1 ^a^	83.0 ± 1.3 ^a^

Means in same column with different superscripts (a, b) are statistically different (*p* < 0.05).

## Data Availability

Data is contained within the article.
